# Supplementary material for: A Multi-Filovirus Vaccine Candidate: Co-Expression of Ebola, Sudan, and Marburg Antigens in a Single Vector
Source: Vaccines (Basel). 2020 May 21;8(2):241. doi: 10.3390/vaccines8020241 (PMC7349952; doi:10.3390/vaccines8020241)
Supplement: Supplementary file 1 [file vaccines-08-00241-s001.pdf]

vaccines
MDPI

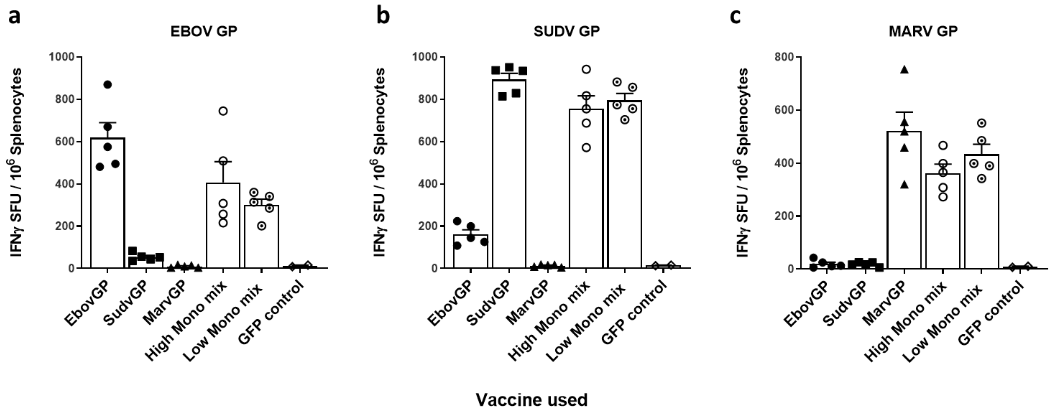


**Figure S1** Immunogenicity and cross reactivity of monovalent ChAdOx1 vaccines administered individually or as a mix.

BALB-c mice received either monovalent vaccines expressing EBOV, SUDV, MARV or GFP or a mix of monovalent controls. High Mono mix consists of $10^8$ IU of each of the three vectors, whereas low mono mix consists of a total of $10^8$ IU across all three vectors (3.33 x $10^7$ IU per vector). IFN-γ ELISpot was performed 2 weeks post vaccination. Splenocytes were stimulated *ex vivo* with peptide pools spanning (a) EBOV, (b) SUDV or (c) MARV glycoproteins. (Data for Low Mono Mix (circles with dots in) is duplicated from Figure 2.)

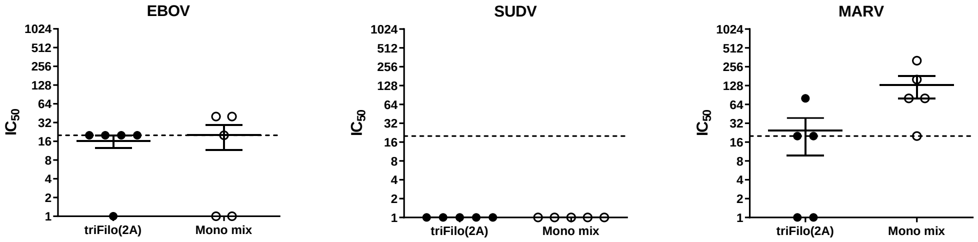


**Figure S2.** Neutralising Antibody levels induced by ChAdOx1 TriFilo Prime.

CD-1 mice were vaccinated with either triFilo(2A) vaccine or mix of monovalent controls. Neutralising antibodies were measured 8 weeks later using pseudotyped lentiviruses expressing the glycoprotein from Zaire Makona strain (15.1), Sudan, or Marburg Angola. Dotted line represents lower limit of detection of the assay.

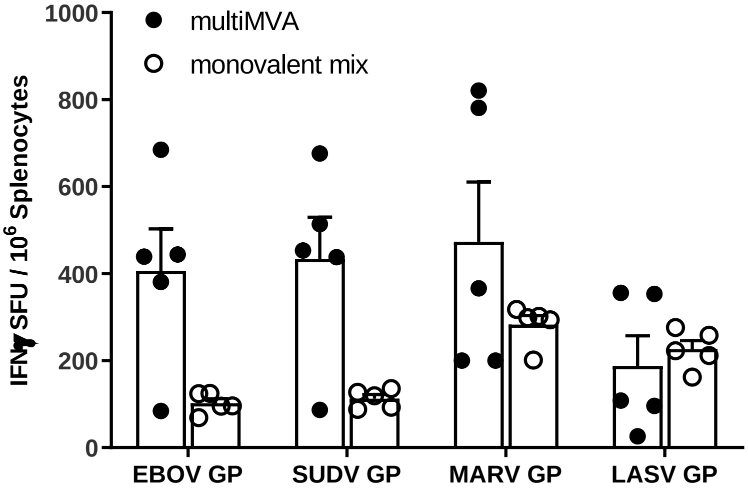


**Figure S3. Cellular Immunogenicity of Monovalent and Multivalent MVA vaccines.** BALB/c mice received either multiMVA or mix of monovalent controls, and IFN-γ ELISpot was performed at 2 weeks post vaccination using peptide pools covering the filovirus glycoproteins shown on the x-axis.

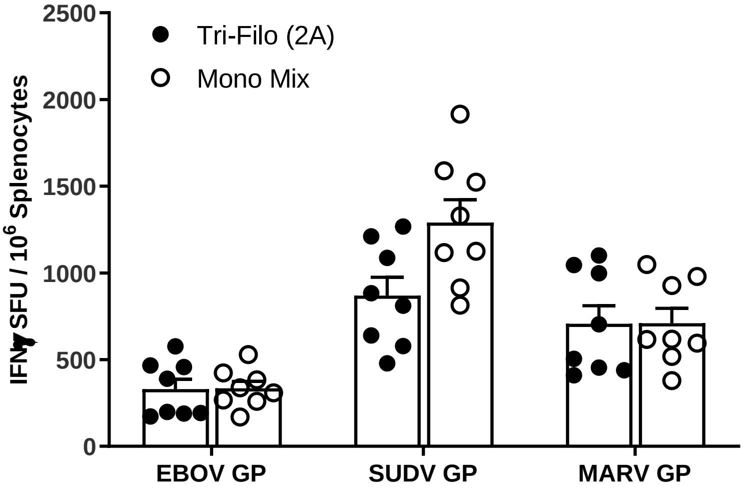


**Figure S4 Cellular Immunogenicity of triFilo(2A) in CD-1 mice.** CD-1 mice received either triFilo(2A) or mix of monovalent controls and IFN-γ ELISpot was performed at 2 weeks post vaccination using peptide pools covering the filovirus glycoproteins shown on the x-axis.

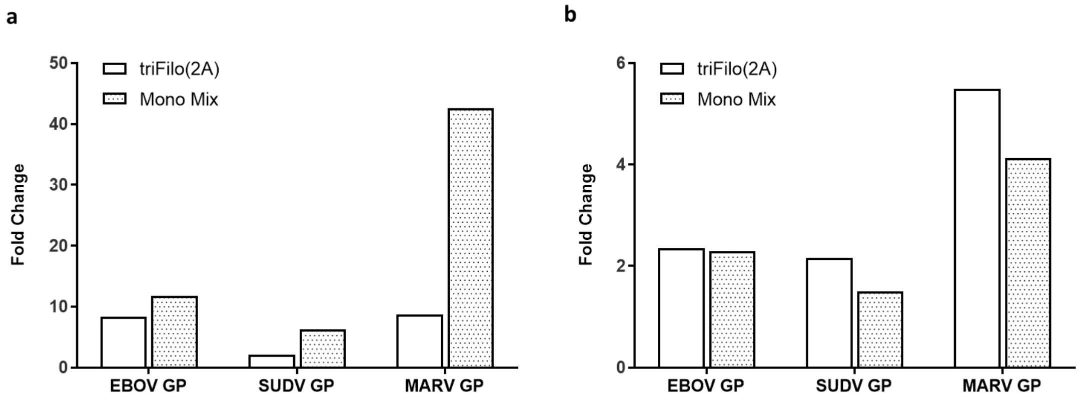


**Figure S5.** Antibody levels induced by ChAdOx1 TriFilo Prime followed by multiMVA boost.

Mice were primed with either triFilo(2A) vaccine or mix of monovalent controls, subsequently all mice were boosted with multiMVA. Fold change of IgG antibody titres, comparing day before boost to post-boost in: a. BALB/c, 10 week prime-boost interval, post-boost timepoint 2.5 weeks later. b. CD-1, 4 week prime-boost interval, post-boost timepoint 8 weeks later

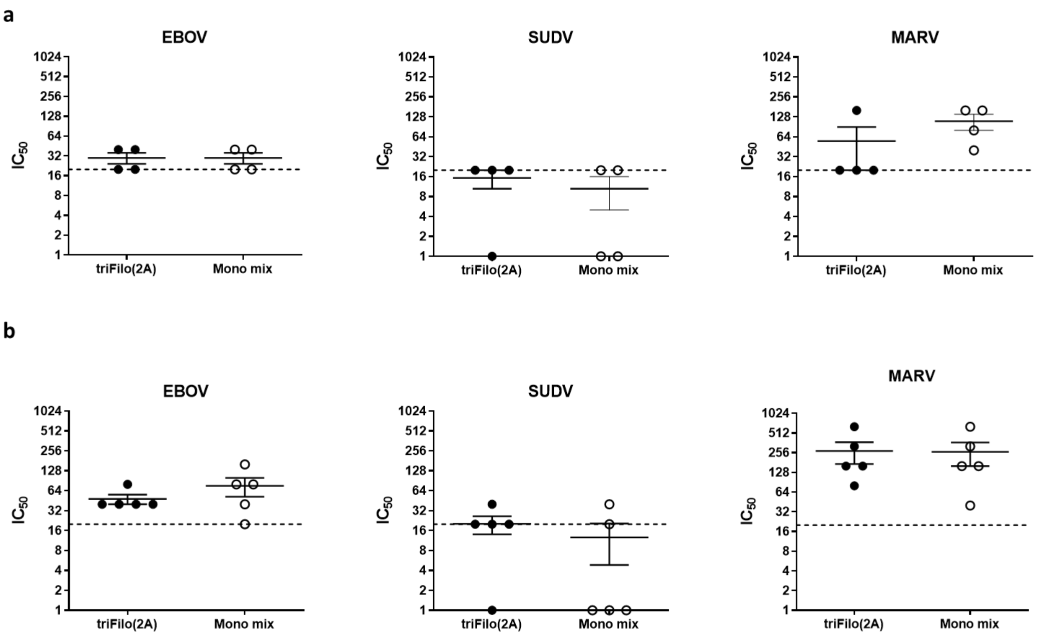


**Figure S6.** Neutralising Antibody levels induced by ChAdOx1 TriFilo Prime followed by multiMVA boost.

Mice were primed with either triFilo(2A) vaccine or mix of monovalent controls, subsequently all mice were boosted with multiMVA. Neutralising antibodies were measured using pseudotyped lentiviruses expressing the glycoprotein from Zaire ebolavirus Makona, Sudan ebolavirus or marburgvirus Angola isolate in 2 mouse strains: a. BALB/c, 21 week prime-boost interval, post-boost timepoint 3 weeks later. B. CD-1, 4 week prime-boost interval, post-boost timepoint 8 weeks later.

Dotted lines represent lower limit of detection of the assay.

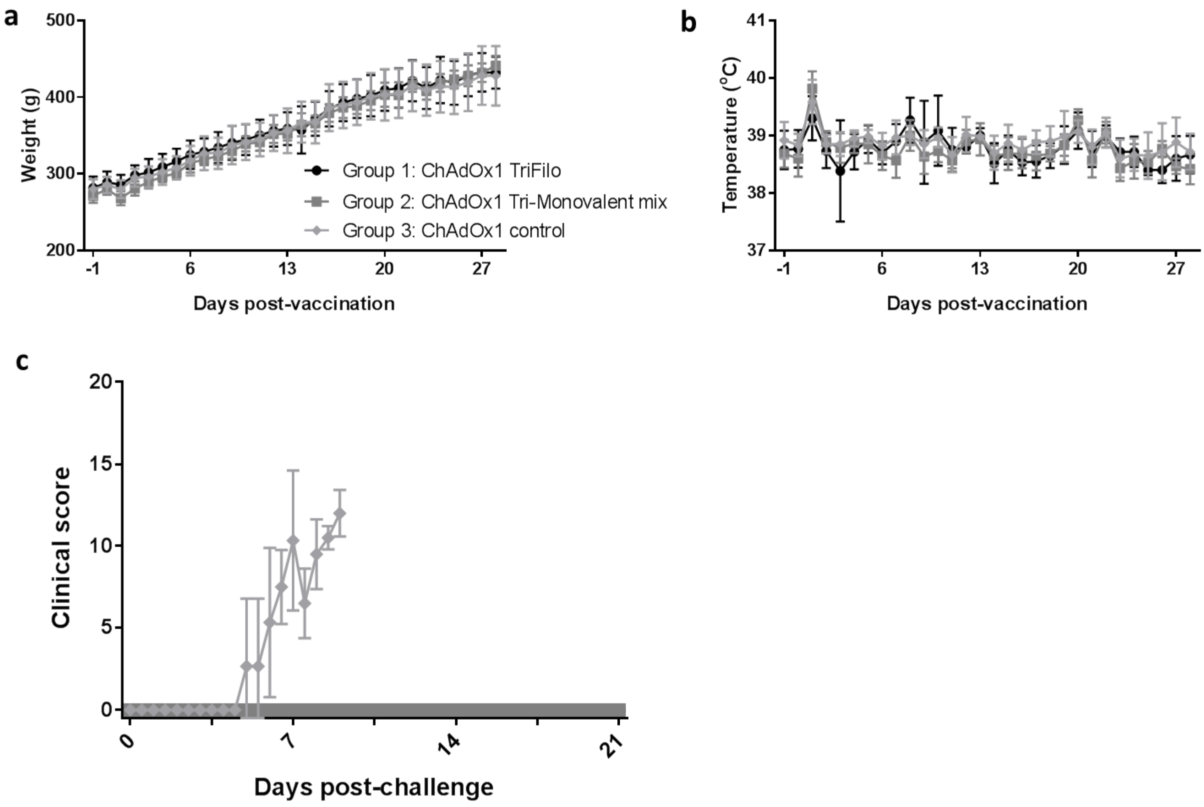


**Figure S7.** Heterologous EBOV Challenge in Guinea Pigs – Additional Data.

(a) Weight and (b) temperature of animals immunised with ChAdOx1 filovirus vaccines compared to control animals receiving ChAdOx1 control. (c) Clinical score of animals immunised with ChAdOx1 filovirus vaccines or ChAdOx1 control after challenge with Ebola virus. Graphs show mean values from up to 6 animals per group with error bars denoting standard error.

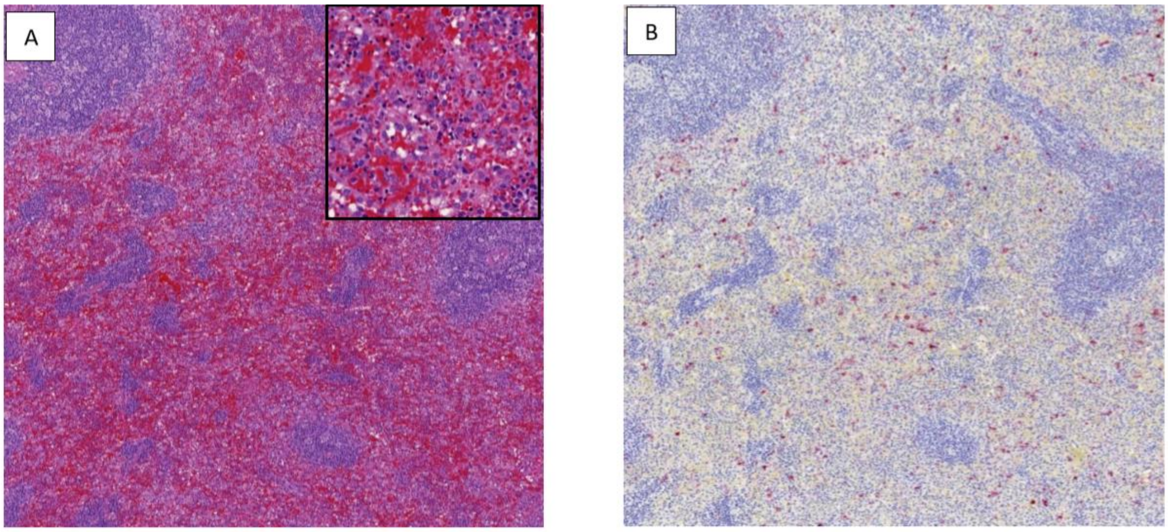

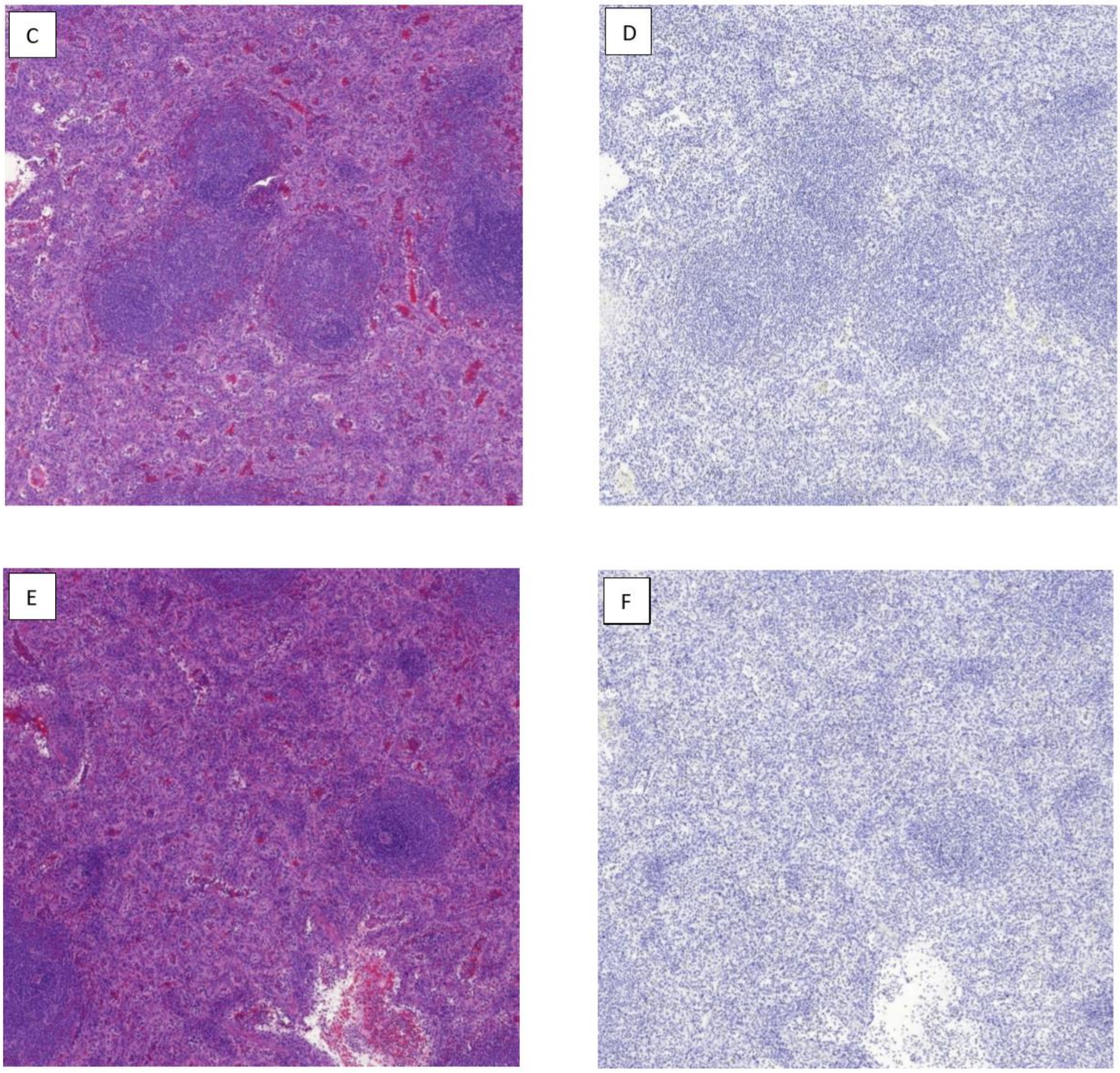


**Figure S8.** Spleen Histology following Heterologous EBOV Challenge in Guinea Pigs.

(a), (c) & (e) haematoxylin and eosin stain, x100 magnification

(b), (d) & (f) *In situ* hybridisation for EBOV viral RNA, which stains pink, x100 magnification

(a) & (b) Images from ChAdOx1 control immunised guinea pig (a) Microscopic changes consistent with infection with Ebola virus; these comprise marked red pulp congestion and scattered, single cell necrosis located randomly throughout the parenchyma. Inset, scattered necrotic cells in the red pulp, x800. (b) Prominent and frequent numbers of cells staining positive for Ebola viral RNA.

(c) & (d) Images from ChAdOx1 TriFilo-2A immunised guinea pig. (c). Normal splenic parenchyma. (d) Absence of staining for Ebola viral RNA.

(e) & (f) Images from ChAdOx1 monovalent mix immunised guinea pig (e) Normal splenic parenchyma. (f) Absence of staining for Ebola viral RNA.

© 2020 by the authors. Submitted for possible open access publication under the terms and conditions of the Creative Commons Attribution (CC BY) license (http://creativecommons.org/licenses/by/4.0/).
